# INTERNET USE, MENTAL HEALTH, SOCIAL SUPPORT, AND CARE BURDEN OF INFORMAL CAREGIVERS

**DOI:** 10.1093/geroni/igz038.499

**Published:** 2019-11-08

**Authors:** Eun-Hye G Yi, Margaret E Adamek

**Affiliations:** Indiana University School of Social Work, Indianapolis, Indiana, United States

## Abstract

Given the psychological stress and health difficulties that stem from caregiver burden and lack of support, various technology-based supports have been introduced. This study aimed to understand the relationships of internet and social network use on informal caregivers’ health status, social support, and care burden. Outcomes were compared for three groups: Alzheimer’s caregivers (AC), other types of caregivers (OC), and non-caregivers (NC). A secondary data analysis of national data from the Health Information National Trends Survey 5-cycle for 2018 was conducted (N=3,297; NC= 2,918, OC=443; AC=113). Using Stata SE 15.1, various statistical analyses (Chi-square, ANOVA, logistic regression) with jackknife bias correction were used to compare the 3 groups. In general, OC experienced the most serious depression/anxiety. More OC (32%) were diagnosed with depression/anxiety by health professionals than NC (22.64%) and AC (19.82%) (F(2,48)=5.58, p<.001). The Other Caregiver group also had higher scores in PHQ-4 (M=2.42, SD=3.07) than NC (M=1.75, SD=2.71) and AC (M=1.63, SD=2.42) (F(2,48)=3.97, p<.05). Regarding care burden, AC provided more support for their care recipients for ADLs (M=2.93, SD=1.65) and IADLs (M=3.75, SD=2.00 for AC) than OC (F(1,49)=4.39, p<.05 for ADLs; F(1,49)=3.17, p<.10 for IADLs). OC (M=2.57, SD=2.43) and AC (2.96, SD=2.39) had fewer social supports than NC (M=3.41, SD=2.38) who can instantly assist them (F=11.60, p<.001). OC (21.68%) and AC (29.00%) more frequently than NC (12.01%) participated in social network (F(2,48)=5.72, p<.01) and online support groups (OC: 13.68%; AC=12.73%; NC=4.68%) (F(2,48)=9.84, p<.001). Developing practical online approaches for informal caregivers can supplement their limited in-person social support.

